# Non-chemical weed management: Harnessing flame weeding for effective weed control

**DOI:** 10.1016/j.heliyon.2024.e32776

**Published:** 2024-06-11

**Authors:** Abhishek Upadhyay, Krishna Pratap Singh, K.B. Jhala, Mohit Kumar, Ali Salem

**Affiliations:** a Department of Farm Machinery and Power Engineering, ICAR – Central Institute of Agricultural Engineering, Bhopal, Madhya Pradesh, 462038, India; b Indian Council of Agricultural Research, New Delhi, 110001, India; c Department of Farm Machinery and Power Engineering, Junagadh Agricultural University, Junagadh, Gujarat, 362001, India; d Sri Karan Narendra Agriculture University, Jobner- Jaipur, Rajasthan, 303329, India; e Civil Engineering Department, Faculty of Engineering, Minia University, Egypt; f Structural Diagnostics and Analysis Research Group, Faculty of Engineering and Information Technology, University of Pécs, Hungary

**Keywords:** Flame weeder, Thermal weed control, Weed control efficiency, Energy consumption, Recovery rate

## Abstract

The goal of the current study was to create and assess the effectiveness of a hand-pulled ergonomically designed flame weeder. The developed weeder was tested in the field at three operating pressures (20, 30 and 40 Psi) and forward speeds (1.00, 1.25 and 1.50 km/h) to study their effects on plant damage, survival rates, weight preservation rates, weed management effectiveness, soil temperatures, and gas and energy consumption. Thereafter, at optimized values of forward speed and operating pressure, a comparative assessment of flame weeding with traditional methods (mechanical and manual weeding) was done in terms of weed control effectiveness, operational time, energy consumption, and cost of operation. Results showed that the optimal performance of the designed flame weeder was achieved when operated at a speed of 1 km/h and an operating pressure of 40 psi. The survival rate, weight preservation rate, weed control efficiency, change in soil temperature, recovery rate, plant damage, gas consumption, and energy consumption were observed to be 27.3 %, 32.5 %, 91.1 %, 40.74 °C, 8.5 %, 2.2 %, 4.05 kg/h, and 2500.24 MJ/ha, respectively, at optimized values of forward speed (1.00 km/h) and operating pressure (40 Psi). The actual field capacity, field efficiency and operating cost of the flame weeder were 0.0755 ha/h, 94.94 %, and 3620.81 ₹/ha, respectively. Hand weeding had the best level of weed control effectiveness, but it was a laborious, time-consuming process. When compared to manual weeding, flame weeding was 50.42 % cheaper and 94.82 % faster.

## 1 Introduction

In a changing socio-economic context, Indian agriculture can only survive with the application of improved technologies in agricultural engineering. There were numerous agricultural issues such as climate change, pests and insects but weeds were one of the main causes of declining yield in Indian agriculture. Weeds were a major constraint in both conventional and organic crop production systems. In order to address this issue, weed scientists around the world were studying alternative weed control methods based on integrated weed control measures to reduce herbicide dependence. There were various weed control methods like chemical, physical, biological, and mechanical. Every year, there is a rise in destructive soil-borne pests such as verticillium, blight, greensickness, root rot, and root-knot nematodes. These pests often reduce yields by 20–60 % and occasionally by more than 60 % [1]. Scientists have recently renewed their interest in flaming fire, i.e., thermal weed control, largely because of recent advances in flame technology. Thermal weed control methods were the best tools used when environmental or health issues were concerned, where non-specific damage to unplanned vegetation is significant and earns extra interest in the integration of unconventional planting systems [2]. Burning has advantages over herbicides such as the absence of chemical residues and weedicide-resistant weeds. Weed control with herbicides is not possible in organic farming. Compared to chemical weed control, the flame weeder offers many benefits as it does not contaminate groundwater, soil, and air.

Mechanical weeding requires a strong frame to plough the soil, which increases the input strength, while flame cultivation does not require high input strength because flame cultivation does not interfere with soil formation.

Flame weeding uses controlled flames to eradicate undesired flora as a sustainable and eco-friendly substitute for traditional weed control techniques. This method has attracted attention because it can lessen the need for chemical herbicides and have a smaller negative impact on the environment. Flame weeding does not pose a risk to human safety in the environment as it does not pose a risk of flooding as chemical treatments. In contrast to chemical weed control, flame weeding provides a way to treat weeds resistant to toxins. Instead of using pesticides, physical techniques of controlling soil-borne nematodes are becoming increasingly popular. The flame sterilization technique or soil flame disinfestation (SFD) uses direct flaming to eradicate an infestation physically. SFD is a creative and non-chemical technique for managing agricultural pests carried through the soil. A flame weeder would be a better option, so designing and developing a flame weeder was decided.

Numerous research works have concentrated on the engineering and design of flame weeding equipment. With multiple fuel sources, burner arrangements, and control methods, innovations extend from portable devices to tractor-mounted systems. Zhang et al. [3] contributed significantly by introducing a precision flame weeder with an automated targeting system, which improved accuracy and efficiency. Other researchers have looked into the use of alternative fuels [4]. The literature emphasizes that to improve the effectiveness of weed management, flame characteristics, nozzle design, and combustion efficiency must be optimized [5,6].

Evaluating variables, including fuel consumption, operational efficiency, and weed mortality rates, determines how well a flame weeder performs. Studies on flame weeding's immediate effects on target vegetation are more common than those that highlight the long-term implications, which can include microbial activity and soil health [7,8]. Studies on flame weeding's effects on the environment by Pressler et al. [9] and Rajkovic et al. [10] highlight the technique's ability to minimize soil disturbance and lower chemical runoff. Specifically, research by Morselli et al. [11] and Mojžiš and Varga [12] has examined optimizing flame intensity and quantifying thermal impact zones for better weed management without sacrificing crop health. The effects of flame temperature and speed on performance and weed fatality rates were also examined [13,14]. The effectiveness of soil flame disinfestation (SFD) against pathogenic pests and nematodes has been established [15,16].

In this field, there are still several knowledge gaps despite progress. The first issue impeding direct comparisons between research is the absence of standardized testing methodologies for flame weeders. Furthermore, nothing is known about how frequent flame weeding affects microbial populations and soil health in the long run. This is an important gap since more extensive ecological effects must be considered for sustainable weed management.

The objective of the manuscript is conveyed by the knowledge gaps that have been found. In order to close these gaps, the planned research would standardize testing protocols, look into the effects of flame weeding on the environment, and adapt the technology to various agricultural environments. The manuscript hopes this will help advance sustainable weed management approaches, which is the larger purpose.

## 2 Materials and methods

The flame weeder was designed and developed at the Farm Machinery and Power Engineering Department workshop, CAET, Junagadh Agricultural University, in the year 2020. The field evaluation was conducted on a research plot of Mechanized Commercial Farm, Department of Agronomy, COA, Junagadh Agricultural University, Junagadh (Gujarat) (20°30′N latitude and 69°41′E longitude) in the summer season in the year 2021. The soil type was clay loam (sand: 23.36 %, silt: 39.64 %, and clay: 37 %). The experimental field was of size 30 × 46 m.

**Fig. 1:**
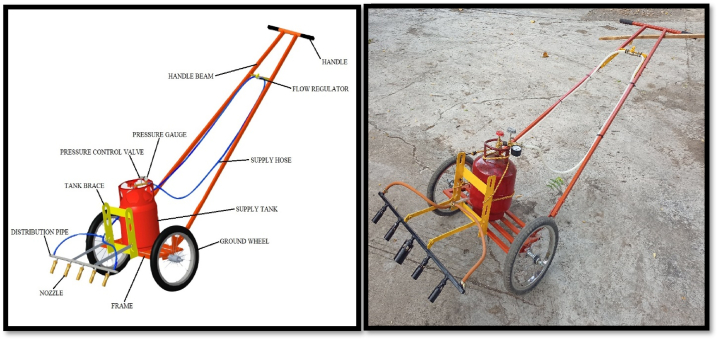
Isometric view of developed flame weeder.

Based on design consideration, the flame weeder was developed (Fig. 1), which consists of the following components (Table 1). This machine was developed by taking all the ergonomic factors into consideration.

**Table 1:** Specifications of the components of the developed machine.

S. No.	Components	Specifications
1	**Handle**	
	Material of construction	G.I. circular pipe
	Length (mm)	500
2	**Handle beam**	
	Length (mm)	1500
3	**Frame**	
	Length (mm)	600
4	**Supply tank**	5 kg
5	**Tank brace**	
	Material of construction	M.S. Flat
6	**Ground wheel**	2
	Overall Diameter (mm)	400
7	**Nozzle assembly**	
	Material of construction	G.I. square pipe
	Length of distribution pipe (mm)	765
8	**Flow Regulator**	1
9	**Pressure Gauge**	1
10	**Nozzle (**Brass Alloy**)**	5

### 2.1 Performance evaluation

Performance evaluation of the developed flame weeder was carried out according to the experimental plan (Table 2). The independent variables had 3 levels, whose values ranged from 1.00 to 2.00 km/h for forward speed because the average speed of humans for any manual work lies in this range and operating pressure of 20–40 psi, as suggested by Spagnolo for flame weeding [17]. The experiments were conducted as per factorial in a completely randomized block design [18]. The best combination of speed and pressure was observed based on different performance parameters. After that, a cost analysis was also carried out. There were nine treatments, and each treatment was replicated thrice.

**Table 2:** Experimental plan.

Independent variables	Levels	Dependent variables
Operating pressure	20 Psi (P_1_), 30 Psi (P_2_), 40 Psi (P_3_)	Survival rate, Weight preservation rate, Weed control efficiency, Change in soil temperature, Recovery rate, Plant damage, Gas consumption, Cost of operation
Forward speed	1.00 km/h (S_1_), 1.25 km/h (S_2_), 1.50 km/h (S_3_)	
Replications	3	
Statistical design	Completely randomized block design	

### 2.2 Determination of performance parameters

The performance parameters include survival rate, weight preservation, weed control efficiency, soil temperature, plant damage, recovery rate, gas consumption, energy consumption, and field efficiency. A blow torch for weeding operation ignited the flame weeder, and a flow regulator regulated the gas flow from the supply tank to the nozzle.

#### 2.2.1 Survival rate

It is the ratio of a number of weeds that survived ten days after applying the flame to the total number of weeds present before applying the flame. It was calculated by equation (1) [19].

(1) $$\text{SR}\left(\%\right)=\frac{\text{Na}}{\text{Nb}}\times 100$$

where,

N_b_ and N_a_ were the number of weeds counted per unit area before and after applying the flame.

#### 2.2.2 Weight preservation rate

Weight preservation rate shows the weight gain achieved by weeds, once treated with flame, at sampling (10 days after flaming). The weight preservation rate was calculated by using equation (2) [19].

(2) $$\text{WPR}\left(\%\right)=\frac{\text{Wa}}{\text{Wb}}\times 100$$

where,

W_b_ is the average weed weight in the control plot, and W_a_ is the average weed weight after applying the flame.

#### 2.2.3 Weed control efficiency

The weed control efficiency is a factor of two dependent variables, survival and weight preservation rates; it represents both factors combined in a single term. Thus, it is an index that combines both parameters. Weed control efficiency (ƞ_w_) was determined using equation (3) [19].

(3) $${ƞ}_{w}\left(\%\right)=\left[1‐\left(\text{SR}\times \text{WPR}\right)\right]\times 100$$

where,

η_w_ is weed control efficiency (%)

#### 2.2.4 Change in soil temperature

Soil temperature was measured before and after treatment using a digital infrared thermometer. The soil temperature was measured to study the effect of change in soil temperature due to flame on soil texture, structure, and chemical composition.

#### 2.2.5 Recovery rate

The recovery rate reflects the rate at which the weed recovers after 7 days of flaming, and observations were taken immediately after operation within 3 h, it calculates the ratio in the percentage of the dry weight of weeds within the unit area from equation (4) [20].

(4) $$R_{r}=\frac{M_{3}‐M_{2}}{M_{2}}\times 100$$

where,

R_r_ is recovery rate (%), M_3_ is dry weight of weed kg/m^2^ after 7 days and M_2_ is dry weight of weed kg/m^2^ within 3 h after treatment.

#### 2.2.6 Plant damage

Plant damage was calculated by observing the number of plants damaged in the experimental plot to the total number of plants in the experimental plot using equation (5) [21].

(5) $$P_{d}=\frac{A}{B}\times 100$$

where,

P_d_ is Plant damage (%), A is the number of plants that were injured in the experimental plot, and B is the total number of existing plants in the experimental plot.

#### 2.2.7 Gas consumption

Gas consumption is the actual gas consumed during the operation, and it depends on the operating pressure and duration of the operation. It was calculated by equation (6) [17].

(6) $$\text{Gas}\;\text{Consumption}\;\left(\text{kg}/h\right)=\frac{W_{1}‐W_{2}}{T}$$

where,

W_1_ is the weight of the tank before the operation (kg), W2 is the weight of the tank after the operation (kg), and T is the total time taken for the operation (hr).

#### 2.2.8 Energy consumption

The flame weeder was manually operated in the experimental field. In flame weeding operation, the total energy consumption of the machine was calculated by adding manual energy expended and fuel energy consumed, which was given by equation (7).

(7) $$E=E_{m}+E_{g}$$

where,

E is total energy consumption (MJ/ha); _Em_ is manual energy expended (MJ/ha), and _Eg_ is fuel energy expended (MJ/ha).

The human energy utilized in the field for flame weeder was evaluated using equation (8) [22].

(8) $$E_{m}=1.96\;N_{m}T_{m}$$

where,

_Nm_ is the number of labor spent on a farm activity, and T_m_ is the useful time spent by labor on a farm activity (h/ha).

The equation (9) calculated the energy consumed by the combustion of LPG:

(9) $$E_{g}=G_{c}\times C_{V}$$

where,

_Gc_ is gas consumption (kg/ha) and _Cv_ is the calorific value of fuel (MJ/kg).

#### 2.2.9 Cost of operation

The cost of operation of the flame weeder in terms of (₹/ha) was calculated considering fixed costs and variable costs involved in the operation. The cost of operation of weeding, performed for each treatment, was worked out by the prevailing input for machinery, fabrication price of implement, and rental wages of operator or laborers if required.

### 2.3 Comparative assessment of flame weeder with conventional practices

A comparison was made by utilizing a completely randomized block design, between flame weeding and conventional procedures such as hand weeding and mechanical weeding in terms of the effectiveness of weed control, the amount of time required for operation, the amount of energy consumed, and the cost of operation. The weeding technique was considered to be the independent variable in this study. The independent variable was comprised of three different levels. At the 1 % and 5 % levels of significance, the analysis was carried out. Tukey's (b) method was used to compare the mean of the various treatments. There were three different treatments, which were referred to as Flame weeding (T_1_), Mechanical weeding (T_2_), and Hand weeding (T_3_). Each treatment was repeated five times.

## 3 Results

During preliminary trials, it was observed that the machine was easy to operate without any risk to workers. The field observations and results obtained at different independent parameters were analyzed statistically and discussed in detail in the supplementary material.

### 3.1 Survival rate

**Fig. 2:**
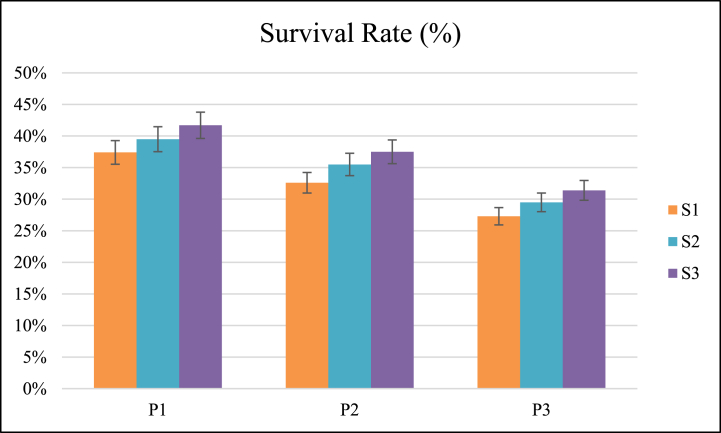
Effect of speed of operation (S) and operating pressure (P) on survival rate.

The survival rate was significantly affected by forward speed and operating pressure. The coefficient of variation of survival rate for different interactions of forward speed and pressure was 13.83 % (Table 3). All three operating pressures and forward speeds were significantly different from each other for survival rate, as shown in Fig. 2.

**Table 3:** Two-way Analysis of variance for different parameters of field evaluation.

Source of variation	Survival Rate (%)	Weight Preservation Rate (%)	Weed Control Efficiency (%)	Recovery rate (%)	Plant damage (%)	Change in soil temperature (°C)	Gas consumption (kg/h)	Energy consumption (MJ/ha)	Cost of operation (₹/ha)
F-value									
Forward speed (S)	12.49^b^	10.732^b^	11.403^b^	29.563^b^	21.188^b^	80.533^b^	314.411^b^	791.820^b^	896.758^b^
Operating pressure (P)	63.64^b^	61.285^b^	60.528^b^	129.655^b^	20.495^b^	180.634^b^	1977.757^b^	1949.567^b^	1833.646^b^
S^a^ P	0.049	0.006	0.098	0.292	0.398	3.690^a^	11.761^b^	31.181^b^	29.440^b^
SE(d)	0.016	0.016	0.012	0.009	0.001	0.97	0.056	44.083	30.715
SE(m)	0.011	0.011	0.008	0.006	0.001	0.69	0.039	31.171	21.719
CV	13.83	11.78	4.07	26.71	15	17.33	38	41.4	34.1

a Significant at 5 % level of significance and.

b Significant at 1 % level of significance.

The maximum and minimum mean value of survival rate was observed as 39.50 % and 29.41 % at pressures of 20 and 40 Psi, respectively. The survival rate decreased with the pressure increase (Table 4). Increasing the operating pressure of gas raises the temperature, affecting the stroma's carbon metabolism and thylakoid lamellae's phytochemical reactions in the chloroplast [23].

**Table 4:** Mean values of parameters affected by operating pressure and forward speed.

Source of variation	Survival Rate (%)	Weight Preservation Rate (%)	Weed Control Efficiency (%)	Recovery rate (%)	Plant damage (%)	Change in soil temperature (°C)	Gas consumption (kg/h)	Energy consumption (MJ/ha)	Cost of operation (₹/ha)
Operating Pressure									
40 Psi	29.41^c^	34.59^c^	89.77^c^	10.60^c^	1.95^b^	38.45^c^	3.50^c^	2784.78^a^	3837.66^a^
30Psi	35.18^b^	39.41^b^	86.07^b^	14.70^b^	1.84^ab^	31.79^b^	2.49^b^	1988.98^b^	2905.49^b^
20 Psi	39.50^a^	44.50^a^	82.36^a^	18.76^a^	1.54^a^	27.83^a^	1.47^a^	1195.54^c^	1977.60^c^
Forward speed									
1.00 km/h	32.40^b^	37.43^a^	87.68^a^	12.76^a^	2.01^a^	36.60^a^	2.87^a^	2499.95^a^	3565.58^a^
1.25 km/h	34.82^ab^	39.50^ab^	86.06^b^	14.64^b^	1.74^ab^	31.91^b^	2.54^b^	1982.14^b^	2890.06^b^
1.50 km/h	36.88^a^	41.57^b^	84.46^c^	16.66^c^	1.58^b^	29.56^c^	2.06^c^	1487.21^c^	2265.12^c^

Numerical in subhead having the same alphabetic superscripts were statistically similar.

The maximum and minimum mean value of survival rate was observed as 36.88 % and 32.40 % at forward speeds of 1.50 and 1 km/h, respectively. The survival rate was increased with an increase in forward speed (Table 4). Decreasing the forward speed increases the effect of temperature on the weed plant, which in turn affects their catalytic activity because prolonged heat stress causes the release of an enzyme, which results in plant cell death [24].

### 3.2 Weight preservation rate

**Fig. 3:**
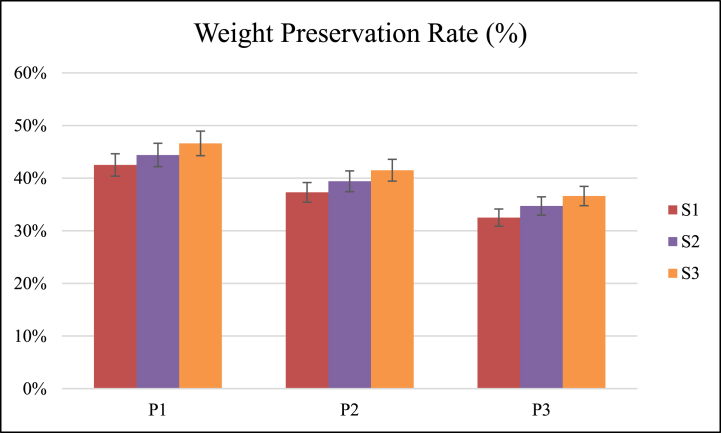
Effect of speed of operation (S) and operating pressure (P) on weight preservation rate.

The weight preservation rate was significantly affected by forward speed and operating pressure. The coefficient of variation of weight preservation rate for different forward speed and pressure interactions was 11.78 % (Table 3). All three operating pressures and forward speeds were significantly different from each other for weight preservation rate, as shown in Fig. 3.

The maximum and minimum mean value of the weight preservation rate was 44.50 % and 34.59 % at pressures of 20 and 40 Psi, respectively. The weight preservation rate was decreased with the increase of pressure (Table 4). Increasing the operating pressure of the gas raises the temperature because temperature rise reduces the biomass of the plants, eventually maturing [25].

The maximum and minimum mean value of weight preservation rate was 41.57 % and 37.43 % at forward speeds of 1.50 and 1 km/h, respectively. The weight preservation rate was increased with an increase in forward speed (Table 4). Decreasing the forward speed raises the temperature, which affects overall photosynthesis, as overgrown plants cause structural damage and long-term inhibition of photosystem II (PSII) activity. The saturation of the reaction centers in the photosystem is increased by thermal stressors, which in turn increases the sensitivity of the system to high irradiance [26].

### 3.3 Weed control efficiency

Weed control efficiency was significantly affected by forward speed and operating pressure. The coefficient of weed control efficiency variation for different forward speed and pressure interactions was 4.07 % (Table 3). All three operating pressures and forward speeds were significantly different from each other for weed control efficiency.

The maximum and minimum mean value of weed control efficiency was observed as 89.77 % and 82.36 % at pressures of 40 and 20 Psi, respectively. The weed control efficiency was increased with the increase in pressure (Table 4). Increasing the operating pressure of the gas raises the temperature, which has an impact on the development and survival of plant species by decreasing seedling growth and reducing plumule/radicle growth [27,28].

**Fig. 4:**
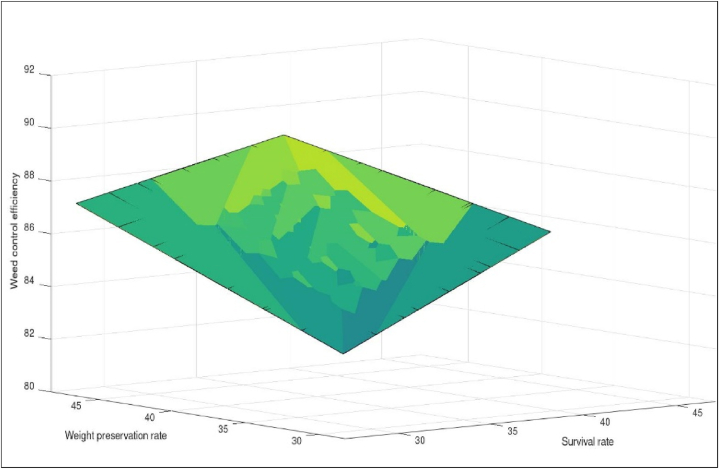
Effect of survival rate and weight preservation rate on weed control efficiency.

The maximum and minimum mean value of weed control efficiency was 87.68 % and 84.46 % at forward speeds of 1.00 and 1.50 km/h, respectively. The weed control efficiency was decreased with an increase in forward speed (Table 4). Decreasing the forward speed affects temperature-regulated inhibitory genes to germination [29]. Weed control efficiency depends on survival and weight preservation rates, as shown in Fig. 4.

### 3.4 Change in soil temperature

Change in soil temperature was significantly affected by forward speed and operating pressure. The coefficient of variation of change in soil temperature for different forward speed and pressure interactions was 17.33 % (Table 3). All three operating pressures and forward speeds were significantly different from each other for changes in soil temperature.

**Fig. 5:**
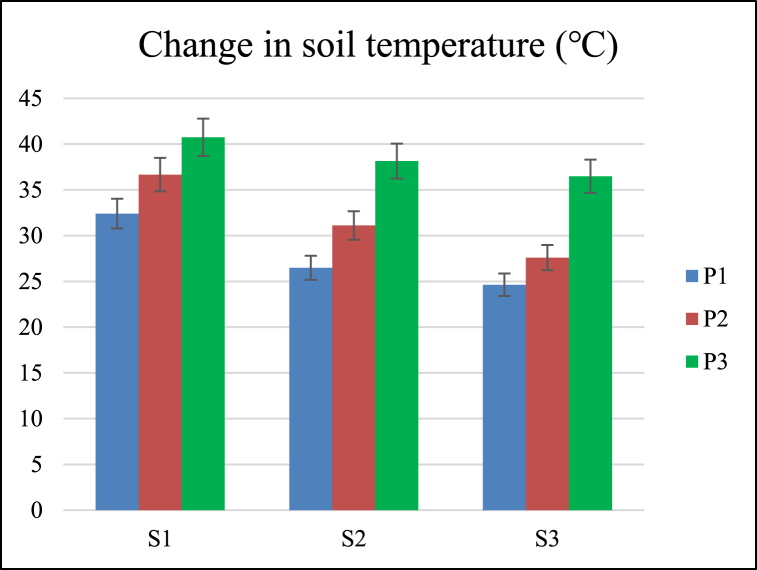
Effect of speed of operation (S) and operating pressure (P) on change in soil temperature.

The maximum and minimum mean values of change in soil temperature were 38.45 °C and 27.83 °C at pressures of 40 and 20 Psi, respectively. The soil temperature was increased with the increase of pressure (Table 4). Increasing the operating pressure of gas raises the temperature, as shown in Fig. 5. A Change in soil temperature over 30 °C increases the aggregate stability of the soil due to the thermal transformation of iron aluminum oxides, which act as cementing agents for clay particles to form strong silt-sized particles in the soil [30]. Root growth was found to rise when the temperature of the soil reached the ideal level. On the other hand, root growth slows down when it reaches excessive levels because increased soil temperature reduces the soil moisture content, maximizes the evaporation rate, and restricts the water movement into the soil profile [31].

The maximum and minimum mean values of change in soil temperature were 36.60 °C and 29.56 °C at forward speeds of 1.00 and 1.50 km/h, respectively. The soil temperature was decreased with an increase in forward speed (Table 4). Decreasing the forward speed raises soil temperature. High temperature alters the soil's physical properties, creating potential risks of subsidence, erosion, and other environmental hazards [32].

### 3.5 Recovery rate

**Fig. 6:**
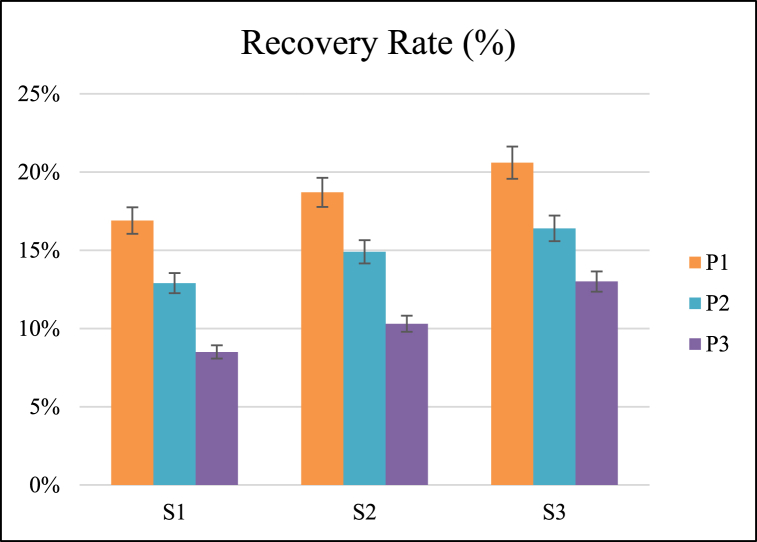
Effect of speed of operation and operating pressure on recovery rate.

The recovery rate was significantly affected by forward speed and operating pressure. The coefficient of variation of recovery rate for different interactions of forward speed and pressure was 26.71 % (Table 3). All three operating pressures and forward speeds were significantly different from each other for recovery rate, as shown in Fig. 6.

The maximum mean value of the recovery rate was observed as 18.76 % for the lowest pressure of 20 Psi, whereas the minimum mean value was observed as 10.60 % for the highest pressure of 40 Psi. The recovery rate decreased with the increase of pressure (Table 4). Heat stress during meiosis is responsible for the sterility of ovules and pollen, in addition to the dehiscence of the anther. Temperatures higher than 30 °C during the formation of pollen cause pollen abortion. Increasing the operating pressure of gas raises the temperature which affects the reproductive development of plants by inhibiting the floral development, fertilization, and postfertilization processes in many plant species [33].

**Fig. 7:**
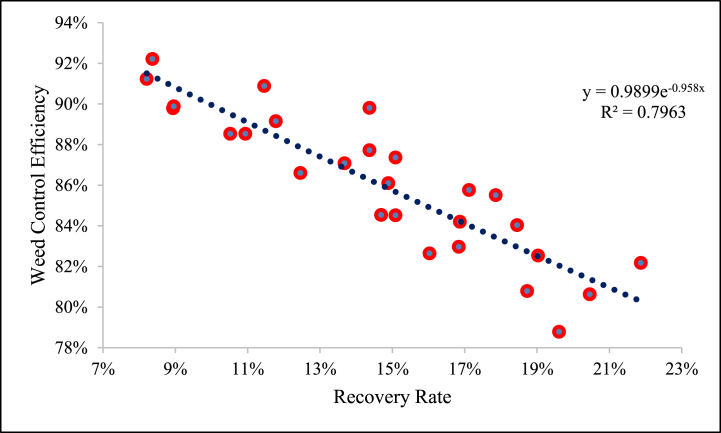
Relationship between weed control efficiency and recovery rate.

The maximum and minimum mean value of recovery rate was observed as 16.66 % and 12.76 % at forward speeds of 1.50 and 1 km/h, respectively. The recovery rate was increased with an increase in forward speed (Table 4). Decreasing the forward speed raises the temperature of the weed plant, relative to opportunity time, affecting plant productivity because a few degrees of temperature during flowering leads to a loss of plant cycle [34,35]. A linear relationship was observed between weed control efficiency and recovery rate, as shown in Fig. 7.

### 3.6 Plant damage

**Fig. 8:**
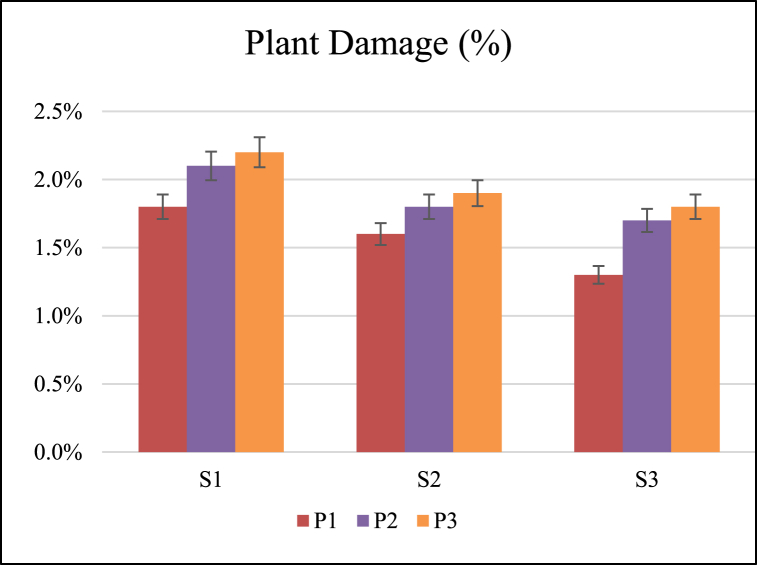
Effect of speed of operation (S) and operating pressure (P) on plant damage.

Plant damage was significantly affected by forward speed and operating pressure as shown in Fig. 8. The coefficient of variation of plant damage for different interactions of forward speed and pressure was 15 % (Table 3). The maximum and minimum mean value of plant damage was observed as 1.95 % and 1.54 % at pressures of 40 and 20 Psi, respectively. The operating pressure was significantly different from the other two pressures for plant damage, while the pressure of 30 Psi and 40 Psi were not significantly different. The plant damage was increased with pressure (Table 4). The maximum and minimum mean value of plant damage was observed as 2.01 % and 1.58 % at forward speeds of 1.00 and 1.50 km/h, respectively. The forward speed of 1.00 km/h was significantly different from the other two speeds for plant damage, while the speeds of 1.25 km/h and 1.50 km/h were not significantly different. The plant damage was decreased with an increase in forward speed (Table 4). Varying the forward speed and pressure of the gas changes the temperature of the crop plant, which affects the opportunity time for lethal heat to reach the crop and the amount of heat that damages the crop. Lower yields are the result of plant reproduction being more susceptible to heat stress than the vegetative stages. This sensitivity impacts several reproductive processes, including stigma receptivity, ovule fertility, pollen germination, pollen load, pollen tube expansion, and seed filling.

### 3.7 Gas consumption

Gas consumption was significantly affected by forward speed and operating pressure. The coefficient of gas consumption variation for different forward speed and pressure interactions was 38 % (Table 3). The maximum and minimum mean value of gas consumption was observed as 3.5 kg/h and 1.47 kg/h at pressures of 40 and 20 Psi, respectively. All three operating pressures were significantly different from each other for gas consumption. The gas consumption was increased with the increase of pressure (Table 4). The maximum and minimum mean value of gas consumption was observed as 2.87 kg/h and 2.06 kg/h for the lowest forward speed of 1.00 and 1.50 km/h, respectively. All three forward speeds were significantly different from each other for gas consumption. The gas consumption was decreased with an increase in forward speed (Table 4).

### 3.8 Energy consumption

Energy consumption was significantly affected by forward speed and operating pressure. The coefficient of energy consumption variation for different forward speed and pressure interactions was 41.47 % (Table 3). The maximum and minimum mean energy consumption values were 2784.78 MJ/ha and 1195.54 MJ/ha at 40 and 20 Psi pressures, respectively. All three operating pressures and forward speeds were significantly different from each other for energy consumption. The energy consumption was increased with the increase of pressure (Table 4). The maximum and minimum mean energy consumption values were 2499.95 MJ/ha and 1487.21 MJ/ha at forward speeds of 1.00 and 1.50 km/h, respectively. The energy consumption was decreased with an increase in forward speed (Table 4). Values of effective field capacity and field efficiency for different forward speeds are shown in Table 5.

**Table 5:** Mean values of parameters affected by forward speed.

Speed	Effective field capacity (ha/h)	Field efficiency (%)
S_1_	0.0575	94.94
S_2_	0.0644	85.91
S_3_	0.0700	77.88
CV	9.78	9.90

### 3.9 Cost of operation

The cost of operation (₹/ha) was significantly affected by forward speed and operating pressure, varying with the effective field capacity. The coefficient of variation of cost of operation for different interactions of forward speed and pressure was 34.10 % (Table 3). All three operating pressures and forward speeds were significantly different from each other in the cost of operation. The maximum and minimum mean value of the cost of operation was observed as 3837.66 ₹/ha and 1977.60 ₹/ha at pressures of 40 and 20 Psi, respectively. The cost of operation was increased with the increase of pressure (Table 4).

**Fig. 9:**
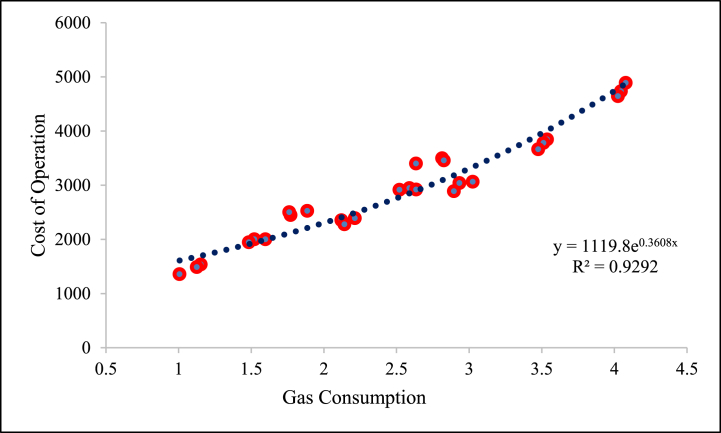
Relationship between gas consumption and cost of operation.

The maximum and minimum mean value of the cost of operation was observed as 3565.58 ₹/ha and 2265.12 ₹/ha at forward speeds of 1.00 and 1.50 km/h, respectively. The cost of operation was decreased with an increase in forward speed (Table 4). Decreasing the forward speed increases the gas consumption per unit area, increasing the operation cost. In contrast, gas consumption increases with pressure, so the cost of operation increases. The relationship between gas consumption and cost of operation is shown in Fig. 9.

### 3.10 Comparative assessment of flame weeder with conventional practices

The performance of different weeding methods was compared in terms of weed control efficiency, operational time, energy consumption, and cost of operation. The performance parameters were evaluated and analyzed to conclude by statistical analysis, in which it was observed that weeding methods had significantly (p < 0.05) influenced the performance parameters (Table 6).

**Table 6:** Analysis of variance of parameters for different methods of weeding.

Source of Variation	Weed control efficiency (%)	Operational time (man-h/ha)	Energy consumption (MJ/ha)	Cost of operation (₹/ha)
F-value				
Weeding Methods	150.832^a^	8998.99^a^	5258.657^a^	1840.653^a^
SEm±	0.818	1.456	17.443	77.869
CD	2.708	4.823	57.769	257.884
CV	2.051	3.382	3.704	2.981

*Significant at 5 % level of significance and.

a Significant at 1 % level of significance.

**Fig. 10:**
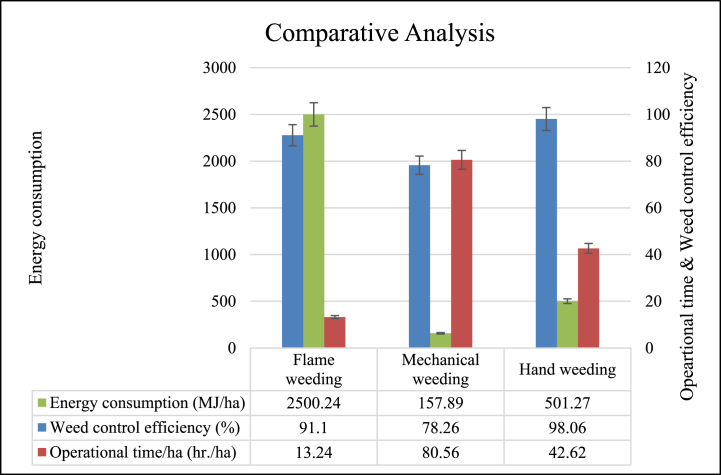
Comparative analysis of flame weeding with conventional weeding practices.

It was revealed from Fig. 10 that the mean value of weed control efficiency was observed to be highest for hand weeding at 98.06 %, followed by the mean value of flame weeding and mechanical weeding at 91.10 % and 78.26 %, respectively but the mean value of operational time was observed to be highest for hand weeding as 255.75 man-h/ha followed by mechanical weeding and flame weeding as 19.84 man-h/ha and 13.24 man-h/ha, respectively. The energy consumption was observed to be highest for flame weeding at 2500.24 MJ/ha, followed by hand weeding and mechanical weeding at 501.27 MJ/ha and 157.89 MJ/ha. Whereas cost of operation was observed to be highest for hand weeding as 9590.79 ₹/ha, followed by flame weeding and mechanical weeding as 4754.62 ₹/ha and 3208.67 ₹/ha, respectively. All three methods of weeding were significantly different in weed control efficiency, operational time, energy consumption, and cost of operation, respectively (Table 7).

**Table 7:** Mean values of parameters for different methods of weeding.

Source of Variation	Weed control efficiency (%)	Operational time (man-h/ha)	Energy consumption (MJ/ha)	Cost of operation (₹/ha)
Flame weeding (T_1_)	91.10^a^	13.24^a^	2500.24^a^	4754.62^a^
Mechanical weeding (T_2_)	78.26^b^	19.84^b^	157.89^b^	3208.67^b^
Hand weeding (T_3_)	98.06^c^	255.75^c^	501.27^c^	9590.79^c^

Numerical in subhead having same alphabetic superscripts were statistically similar.

## 4 Discussion

Mainly in weed management, a flame weeder's development and performance assessment constitute a noteworthy breakthrough in agricultural technology. Several important factors, such as survival rate, weight preservation rate, weed control efficiency, change in soil temperature, recovery rate, plant damage, and gas and energy consumption, were examined in this study with three forward speeds and three operating pressures.

### 4.1 Effect of forward speeds

The study investigates the relationship between the flame weeder's primary performance metrics and three forward speeds. The findings show that changes in forward speed have a major impact on weed control's effectiveness and survival rate. Reduced forward speeds typically result in more effective weed treatment, most likely by maximizing the weeds' exposure to the flame. There is a trade-off, too, given that high speeds could increase survival rates.

### 4.2 Effect of operating pressures

Similarly, examining three working pressures sheds light on how the flame weeder functions. It was discovered that changes in operating pressure impact plant damage, energy and gas consumption. Increased gas and energy consumption are typically the result of higher pressures, which enhances the efficiency of the weed control procedure. But there's also a higher chance of plant harm associated with this.

### 4.3 Interplay of parameters

The study's mechanistic information clarifies the complex relationships between the input and output factors. For example, a decrease in weed control efficacy is correlated adversely with increased forward speed, although this may result in lower survival rates. Furthermore, the flame weeder's environmental and economic sustainability are directly impacted by the operating pressure's effects on gas and energy consumption.

### 4.4 Survival rate and weed control efficiency

The flame weeder's forward speed and operating pressure are important factors in deciding how long undesired vegetation will survive. Our results imply that more effective weed management occurs at lower forward speeds when combined with an ideal operating pressure. Reduced pace lengthens the flame's contact time with the weeds, increasing the mortality rate of unwanted plants.

**Fig. 11:**
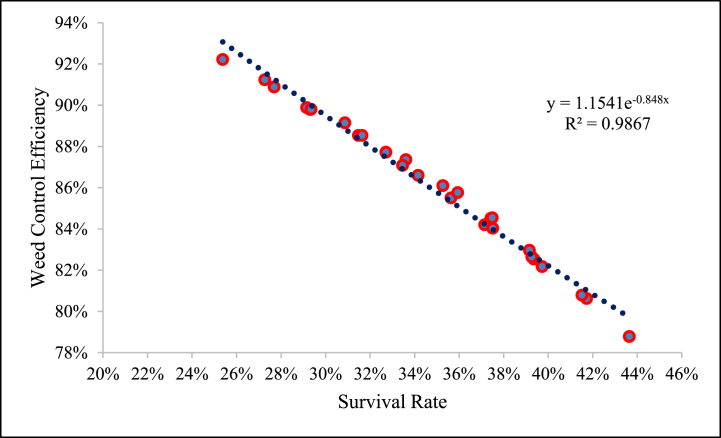
Relationship between survival rate and weed control efficiency.

Hormone homeostasis is one among the numerous physiological and metabolic changes brought on by heat stress during vegetative growth. Fig. 11 illustrates the correlation between the survival rate of weeds and the effectiveness of weed control. This finding is consistent with earlier studies on flame-weeding techniques [36].

### 4.5 Weight preservation rate and recovery rate

One important way to measure how the flame weeder affects the unwanted plants is to look at the target weeds' weight preservation rate. According to our research, this rate highly depends on the forward speed and operating pressure selected. Adjusting these factors can achieve a combination of maximal influence on the weed's weight preservation rate and effective weed control. The primary reason for the changes in membrane functions caused by high temperatures is the change of membrane fluidity. Furthermore, a reduced recovery rate is observed when the ideal ratio of pressure to speed is kept constant.

### 4.6 Plant damage

The relationship between forward speed and operating pressure is essential for limiting unintentional harm to cultivated crops, according to the examination of plant damage. According to our research, these parameters can be carefully adjusted to significantly lessen plant damage while maintaining the general health and yield of the crops.

### 4.7 Change in soil temperature

The effect of the flame weeder on soil temperature is an important factor to consider for agricultural purposes. Our research shows that changes in operating pressure and forward speed result in differences in soil temperature. Lower speeds often result in higher soil temperatures, but operating pressure has a moderating effect. These factors must be calibrated correctly to avoid negative impacts on soil fertility and structure [37].

### 4.8 Gas and energy consumption

One of the most important components of sustainable agriculture practices is energy and gas consumption efficiency. Our findings suggest that the gas and energy consumption of the flame weeder can be affected by carefully choosing the forward speed and operating pressure. Ideal conditions support resource conservation and improve the effectiveness of weed control, which is in line with the increased emphasis on sustainable agricultural methods [38].

## 5 Conclusions

The developed flame weeder was adequate for better weed control. Based on the analysis, the optimum performance for the developed flame weeder was observed at a forward speed of 1.00 km/h and operating pressure of 40 psi. The findings indicated that survival, weight preservation, and recovery rates followed a decreasing trend with increased operating pressure and decreased forward speed. The average weed control efficiency, change in soil temperature, gas, and energy consumption increased with an increase in operating pressure and a decrease in forward speed due to prolonged heat stress to weed plants. The weed control efficiency of flame weeding was 7.63 % less than that of hand weeding, as hand weeding was highly selective and precise. Whereas, it was 16.40 % higher as compared to mechanical weeding. The operational time for flame weeding was 83.56 % and 94.82 % less than mechanical and hand weeding. Flame weeding saves more labor in comparison to mechanical and hand weeding. The cost of operation for flame weeding was 50.42 % less than that of hand weeding but 49.51 % more than that of mechanical weeding. It can be concluded that flame weeding was more feasible than conventional practices and saved time, labor, and cost.

## 6 Future scope

New engineering technologies, including sensors for instantaneous feedback and control algorithms for precision, will be used to improve and optimize the flame weeder design. Long-term environmental and financial implications of mass flame weeder use need further study. Integrating digital technology like machine learning algorithms may yield unique solutions for self-governing systems and adaptive weed control methods. This research lays the groundwork for rapid innovation and the long-term development of flame-weeding technologies in modern agriculture.

## Funding

This research received no external funding.

## Data availability

The data supporting the findings of this study are not deposited in a publicly available repository. However, the data will be made available upon reasonable request. Interested researchers may contact the corresponding author for access to the data.

## Ethical approval

Not applicable.

## Consent to participate

Not applicable.

## Consent to publish

Not applicable.

## CRediT authorship contribution statement

**Abhishek Upadhyay:** Writing – original draft, Software, Methodology, Formal analysis, Data curation, Conceptualization. **Krishna Pratap Singh:** Writing – review & editing, Visualization, Investigation. **K.B. Jhala:** Visualization, Supervision, Software, Resources. **Mohit Kumar:** Visualization, Supervision, Software, Resources. **Ali Salem:** Writing – review & editing, Visualization, Validation, Project administration, Funding acquisition.

## Declaration of competing interest

The authors declare that they have no known competing financial interests or personal relationships that could have appeared to influence the work reported in this paper.
